# Breast Cancer Subtypes Present a Differential Production of Reactive Oxygen Species (ROS) and Susceptibility to Antioxidant Treatment

**DOI:** 10.3389/fonc.2019.00480

**Published:** 2019-06-07

**Authors:** Fabiola Lilí Sarmiento-Salinas, Alam Delgado-Magallón, José Benito Montes-Alvarado, Dalia Ramírez-Ramírez, Juan Carlos Flores-Alonso, Paulina Cortés-Hernández, Julio Reyes-Leyva, Irma Herrera-Camacho, Maricruz Anaya-Ruiz, Rosana Pelayo, Lourdes Millán-Pérez-Peña, Paola Maycotte

**Affiliations:** ^1^Centro de Investigación Biomédica de Oriente, Instituto Mexicano del Seguro Social, Puebla, Mexico; ^2^Posgrado en Ciencias Químicas, Benemérita Universidad Autónoma de Puebla, Puebla, Mexico; ^3^Departamento de Bioquímica, Instituto Tecnológico de Acapulco, Acapulco de Juárez, Mexico; ^4^Centro de Química, Instituto de Ciencias, Benemérita Universidad Autónoma de Puebla, Puebla, Mexico; ^5^CONACYT-Centro de Investigación Biomédica de Oriente, Instituto Mexicano del Seguro Social, Puebla, Mexico

**Keywords:** breast cancer, ROS, mitochondria, mitochondrial morphology, mitochondrial ROS

## Abstract

Due to their crucial role in cell metabolism and homeostasis, alterations in mitochondrial biology and function have been related to the progression of diverse diseases including cancer. One of the consequences associated to mitochondrial dysfunction is the production of reactive oxygen species (ROS). ROS are known to have a controversial role during cancer initiation and progression and although several studies have tried to manipulate intracellular ROS levels using antioxidants or pro-oxidation conditions, it is not yet clear how to target oxidation for cancer therapy. In this study, we found differences in mitochondrial morphology in breast cancer cells when compared to a non-tumorigenic cell line and differences in mitochondrial function among breast cancer subtypes when exploring gene-expression data from the TCGA tumor dataset. Interestingly, we found increased ROS levels in triple negative breast cancer (TNBC) cell lines and a dependency on ROS for survival since antioxidant treatment induced cell death in TNBC cells but not in an estrogen receptor positive (ER+) cell line. Moreover, we identified the mitochondria as the main source of ROS in TNBC cell lines. Our results indicate a potential use for ROS as a target for therapy in the TNBC subtype which currently has the worst prognosis among all breast cancers and remains as the only breast cancer subtype which lacks a targeted therapy.

## Introduction

Breast cancer is a highly heterogeneous disease whose classification has proven to be central for proper patient management, follow-up, clinical trial selection and focus on translational research (1). Breast cancer classification has gradually shifted from a classification based on morphological findings into a more integrative approach which incorporates tumor biomarkers and molecular information. About 75% of breast tumors express the estrogen (ER) and/or progesterone (PgR) receptors and can be targeted with endocrine therapy. Hierarchical clustering of gene expression data of several tumors has led to the definition of molecular intrinsic tumor subgroups, classifying most ER+ tumors in the luminal subtype due to the expression of genes characteristic of luminal epithelial cells (2, 3). Patients with a low risk of relapse are found in the Luminal A subtype while patients in the Luminal B subgroup have a higher risk of relapse and their tumors express increased proliferation-related markers (1, 4, 5). More recently, integrative cluster classification based on DNA rearrangement patterns from whole genome sequencing data have further characterized ER+ tumors into 9 different subtypes with differences in clinical outcomes (1). About 10–15% of breast cancers over-express the HER2/erb2/neu receptor protein, a receptor tyrosine kinase that signals cellular proliferation and patients with HER2+ tumors used to have one of the worst prognoses until the advent of anti-HER2 targeted therapies (4). Molecularly, most of the HER2 enriched tumors are HER2+ by immunohistochemistry (4); and HER2+ tumors have been shown to have different combinations of mutations, supporting the existence of subclasses of HER2+ tumors and also indicating a high heterogeneity within this subgroup (1, 5). Finally, triple-negative breast cancer (TNBC), also classified as basal-like breast cancer, is defined by the absence of ER, PgR, and HER2 receptors and thus lacks a targeted therapy. Only chemotherapy options are available for this breast cancer subtype which has the worst prognosis in all cancer stages and also shows a great intrinsic diversity (6–8). Gene expression patterns have led to the identification of 6 different TNBC subgroups (6) and TNBC tumors show multiple copy number alterations affecting most of the chromosomes (1). Since basal-like breast cancers are identified by gene expression profiling and TNBC are characterized by analyzing the absence of receptors by immunohistochemistry, both terms are not strictly synonyms. It is known that approximately 25% of TNBC are not basal-like on gene expression, but it has also been shown that the TNBC phenotype enriches for basal-like cancer (9). Since TNBC cell lines used in this study have been defined as basal (10), both terms (TNBC and basal) are used for TNBC cell lines in this work.

So, despite recent advances in the classification of breast cancer that have led to effective targeted therapies for most patients, evidence suggests that there is a high heterogeneity in breast tumors even among the ones belonging to the same subtype and that patients would benefit the most from a precise classification and a targeted therapy for each individual tumor. On the other hand, finding targetable biological features for each breast cancer subtype has proven to be successful for ER(+)/luminal and HER2(+) patients (8), underscoring the need to find an effective, targeted therapy for TNBC patients.

One of the hallmarks of cancer cells is the de-regulation of cellular energetics in order to fuel cell growth and division (11). Otto Warburg first observed this anomaly in cancer cells which had a high glycolytic activity even in the presence of oxygen and proposed this metabolic shift to be a cancer driver. However, although multiple oncogenes commonly activated in cancer are known to activate glycolysis, they have been shown to also activate mitochondrial metabolism (12). These metabolic changes have brought attention to the role of mitochondria in tumorigenesis and tumor progression but there seems to be no simple explanation for the role of mitochondria in cancer. Instead, mitochondrial functions have been found to vary depending on genetic, environmental and tissue-of-origin differences between tumors (13). One of the characteristics associated with mitochondrial dysfunction is the production of ROS and sensitivity to ROS-induced apoptosis. In this regard, increased ROS have been found in diverse types of cancer and it has been suggested that increased ROS levels in non-transformed cells or in cancer cells could have pro-tumorigenic effects by damaging nucleic acids and promoting genomic instability. However, there is controversy in the literature regarding the role of ROS in tumor progression. While some studies indicate that ROS in cancer cells can activate pro-tumorigenic signaling pathways (14–16), other studies have shown that treatment with anti-oxidants accelerated tumor growth, metastasis and decreased survival in mouse models of cancer (17, 18). In this work, we studied differences in mitochondrial dynamics as well as in the production of ROS in breast cancer cell lines belonging to different subtypes of the disease with the purpose of identifying differences in mitochondrial-dynamics or ROS-related biomarkers which could work as molecular targets for therapy or lead to a better classification of the disease.

## Materials and Methods

### Hierarchical Clustering and Principal Component Analysis

Mitochondria-related genes were obtained from GSEA (19, 20) (mitochondria, OXPHOS signatures) and genes related to mitophagy and mitochondrial dynamics were added for a total of 167 different probes (Supplementary Table 1). ROS-related genes were selected from GSEA (GO_OXIDATION_REDUCTION_PROCESS; ANTIOXIDANT_ACTIVITY;andREACTOME_BIOLOGICAL_OXIDATIONS) as well as from a previously published ROS-signature and complemented with NOX-related genes for a total of 370 different probes (21) (Supplementary Table 1). Gene expression data was obtained from cbioportal.org using mRNA Expression Z scores from The Cancer Genome Atlas (TCGA); Nature, 2012 study (7). Molecular subtype classification in this sample set was performed according to the PAM50 gene signature assay. Samples with mutations were excluded from the mRNA expression analysis and after elimination of non-classified samples or non-available values, 518 samples were analyzed and Pearson hierarchical clustering as well as principal component analysis (PCA) was performed using Expander7 software (22).

### Cell Culture

Breast cancer cell lines were cultured in the following media: MCF10A (DMEM/F12, Caisson DFP18-1LT, 5% horse serum, 0.5 μg/mL hydrocortisone, 20 ng/mL EGF, 100 ng/mL cholera toxin, 10 μg/mL insulin); MCF7 (Eagle's MEM, Caisson MEP-10X1LT, 10 μg/mL insulin, 10% fetal bovine serum, FBS); T47D (RPMI-1640, Caisson, RPP10-10XLT 7.5 μg/mL insulin, 10% FBS); MDAMB231 (DMEM/F12, 10% FBS); MDAMB468 (DMEM/F12, 10% FBS); BT549 (RPMI-1640, Caisson RPP10-10XLT, 7.5 μg/mL insulin, 10% FBS).

### Mitotracker Labeling and Mitochondrial Classification

Mitochondria were labeled with Mitotracker Red CMXRos (ThermoFisher Scientific, M7512). Since fixation is known to disrupt the mitochondrial network (23), we used live cells for mitotracker labeling. Briefly, 100,000 cells were plated on coverslips and after 24 h stained with 250 nM Mitotracker Red in culture medium at 37°C, protected from light. After incubation, cells were washed twice, first with pre-warmed, serum free medium and then with complete medium. Live cells were mounted with 10 μl complete medium and immediately observed on a Zeiss Observer.Z1 microscope equipped with an Axiocam MRm camera and an Apotome illumination system with a 63X oil immersion objective. Cells were classified as completely tubular (I), tubular with some fragments (II), fragmented with some tubules (III), or completely fragmented (IV) as shown in **Figure 2B** by two independent observers.

For mitochondrial ROS labeling, cells grown in coverslips were incubated with 2 μM Mitosox Red (ThermoFisher Scientific, M36008) and 200 nM Mitotracker green (ThermoFisher Scientific, M7514) for 15 min in complete medium, washed with 1X PBS, mounted with 10 μl complete medium and immediately observed on a Zeiss Observer.Z1 microscope equipped with an Axiocam MRm camera and an Apotome illumination system with a 63X oil immersion objective.

### ROS Measurement

ROS were evaluated by fluorescence microscopy and flow cytometry. For microscopy, 30,000 cells were plated in 24-well-plates and, after 24 h, stained with 10 μM dihydroethidium (Sigma Aldrich, D7008-10MG) in culture medium for 30 min at room temperature, protected from light. After incubation, cells were washed three times, first with pre-heated complete medium and then with PBS, fixed and stained with Hoechst. Stained cells were observed with 1 ml of PBS in a Zeiss Observer.Z1 microscope. ROS quantification was performed by flow cytometry. Briefly, 100,000 cells were plated on 6 well-plates and after 24 h stained with 10 μM DHE as previously described. After incubation, cells were washed tree times, first with pre-warmed complete medium and then with PBS, trypsinized and centrifuged at 2,500 rpm. The pellet was resuspended in PBS with 3% FBS for immediate analysis in a BD FACS Canto II flow cytometer. Graphs show mean fluorescence intensity minus autofluorescence control. For ROS^high^ and ^low^ populations, cells were analyzed according to the flow cytometry pipeline shown in Supplementary Figure 3 using Flow Jo V 10.0 software. For mitochondrial ROS evaluation cells were plated as for DHE staining but stained with 5 μM MitoSox Red in pre-warmed medium for 15 min at 37°C. After incubation, cells were washed twice, first with pre-warmed complete medium and then with PBS, trypsinized and centrifuged at 2,500 rpm, the pellet was resuspended in PBS with 3% FBS for immediate analysis in a BD FACS Canto II flow cytometer. Graphs show mean fluorescence intensity minus autofluorescence control.

### Proliferation and Cell Death Assays

Cell proliferation was assessed in a live-cell Incucyte ZOOM System. Cells were plated at a density of 3,000–5,000 cells per well and after 24 h, treated with hydrogen peroxide (H_2_O_2_) or N- acetylcysteine at the indicated concentrations and imaged every 4 h for 24 h. Proliferation was evaluated using the Incucyte software and expressed as % confluency. Cell death was evaluated after 24 h with 10 μM propidium iodide (PI) staining for 10 min. Fluorescence images were taken in the Incucyte ZOOM system and cell death was expressed as % red (PI+) confluency/% total confluency.

### Reagents

All reagents were purchased from Sigma Aldrich unless otherwise specified.

### Statistical Analysis

Graphs show three or more independent experiments and every figure shows the mean ± standard error. *T*-tests, ANOVA and *post-hoc* tests were performed using GraphPad Prism 5 software. Tukey was performed when means were compared to every other mean and Dunnett's *post-hoc* was used for multiple-to one comparison in **Figure 4**. For two group mean comparison performed in **Figure 4A**, a student *t*-test was used.

## Results

### Mitochondrial Functional Status May Reveal Association With Breast Cancer Intrinsic Subtypes

**Figure 1:**
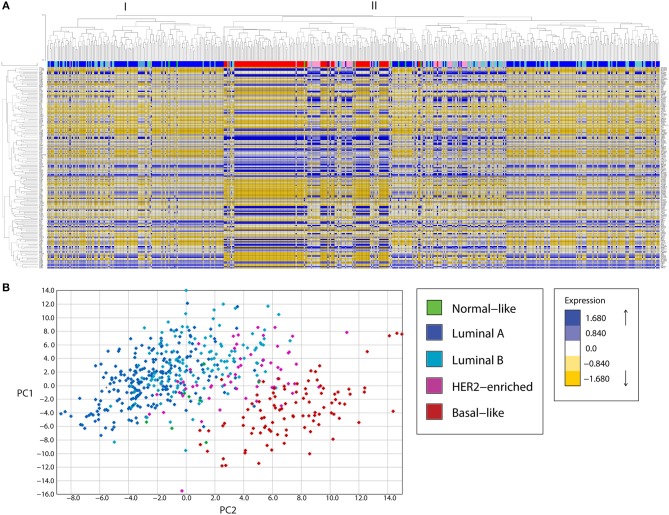
A mitochondria-related gene signature separates breast cancer tumor samples in clusters according to the tumor subtype, in a bioinformatic analysis of gene expression. **(A)** A mitochondria-related gene expression signature was analyzed in TCGA breast tumor samples using unsupervised hierarchical clustering analysis, revealing clusters of samples enriched in molecular subtypes. Two major clusters were found (I, II). Cluster I was enriched in luminal samples while cluster II showed two major sub-groups. The first subgroup was enriched in basal and HER-2 enriched samples while the other was heterogeneous with one sub-group enriched in luminal samples and another containing luminal B and HER2-enriched tumors. **(B)** Principal component analysis (PCA) revealed three main clusters, a cluster of Luminal A and B tumors (left), an intermediate group composed of HER2-enriched and some Luminal B samples and a Basal-like enriched cluster (right). PC, principal component.

Expression analysis of mitochondria-related genes in tumor samples from the TCGA dataset revealed clusters of samples related to molecular breast cancer subtypes, both when analyzed by unsupervised hierarchical clustering (Figure 1A) or principal component analysis (PCA, Figure 1B). Hierarchical clustering analysis revealed two major clusters (I and II). Cluster I was enriched in luminal samples while cluster II was more heterogeneous and two big sub-groups were observed. The first subgroup in cluster II was enriched in Basal-like tumors, which clustered together with HER2-enriched and some luminal samples. The other sub-group in cluster II had a small basal-like cluster, a HER-2 enriched cluster, a luminal B-enriched one and a big luminal cluster containing both Luminal A and B samples. Importantly, luminal tumors in cluster II, were more similar to basal and HER2-enriched tumors than to the other luminal samples in cluster I, evidencing Luminal tumors with potential differences in mitochondrial biology and function.

Mitochondrial differences in breast cancer subtypes were more evident in a PCA analysis (Figure 1B) where a cluster of luminal A and B tumors was found to the left of the graph, followed by an intermediate group with HER2-enriched and luminal B samples and a well-defined Basal-like enriched cluster to the right of the graph which also included some HER2-enriched samples. Thus, PCA analysis of the expression of mitochondria-related genes clustered tumor samples not only according to breast cancer subtypes but also according to malignancy, with the most malignant triple negative or basal-like subtype to the right and the least malignant luminal A samples to the left of the graph (Figure 1B). These findings suggest important differences in mitochondrial function among tumors from different breast cancer subtypes. Importantly, luminal B samples were the most heterogeneous, with some samples clustering with Luminal A tumors (Figure 1B and also Figure 1A, cluster I) and others clustering with HER2-enriched tumors (Figure 1B and also Figure 1A, cluster II, sub-cluster vi). This likely reflects differences in the mitochondrial biology of Luminal B samples which are HER2+ and those which are HER2- and suggests a possible role for the HER2 receptor in the regulation of mitochondrial gene expression and function.

**Figure 2:**
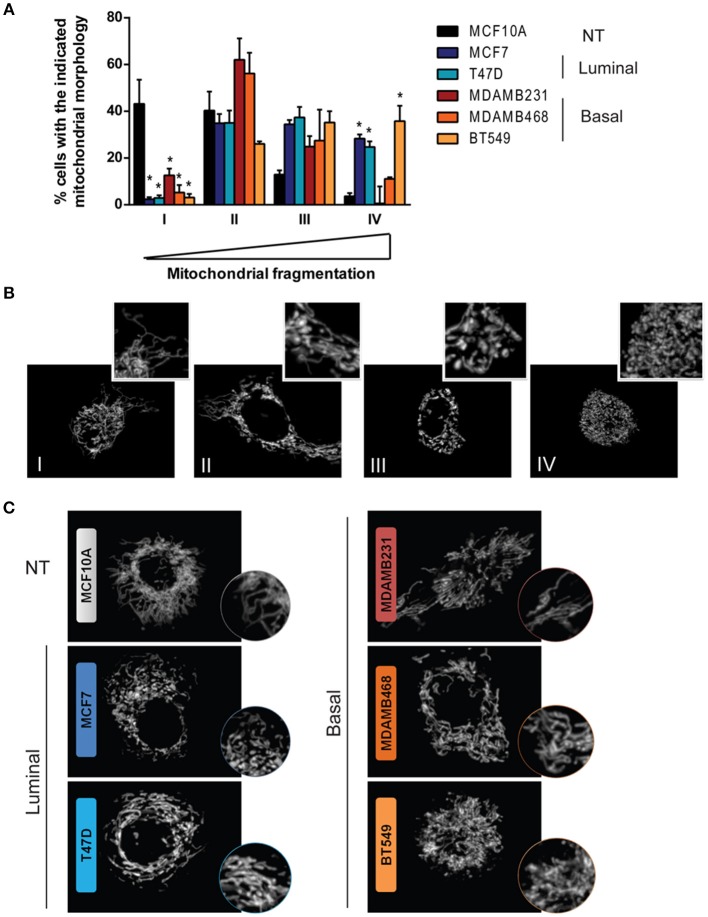
Breast cancer cell lines show differences in mitochondrial morphology and increased mitochondrial fragmentation than a non-tumorigenic cell line. **(A)** Mitotracker staining revealed differences in mitochondrial morphology among breast cancer cell lines. **(B)** Cells were classified as completely tubular (I), tubular with some fragments (II), fragmented with some tubules (III), or completely fragmented (IV). The graph in **(A)** shows the percentage of cells with the corresponding mitochondrial morphology as shown in **(B)**. In **(C)** representative images of the characteristic morphology per cell line is shown. The graph in **(A)** shows mean ± SEM of 3–5 independent experiments. Sixty to one hundred individual cells were classified per experiment by two independent observers. *Different to MCF10A with *p* < 0.05.

Mitochondrial shape has been extensively linked to mitochondrial function and although it is determined by a highly dynamic and regulated process, diverse cellular functions and alterations have been associated to changes in mitochondrial morphology (12, 24). Fluorescent mitochondrial labeling has been used to assess mitochondrial shape and changes in function in breast cancer cells and in cancer cells from other tissues (23, 25–27). Hence, we evaluated mitochondrial morphology in mitotracker-stained breast cancer cell lines representative of different subtypes (Figure 2A). We used MCF10A cells as a non-tumorigenic (NT) control and, according to the classification by Neve et al. (10), we used MCF7 and T47D cell lines as luminal cells and MDAMB231, MDAMB468, and BT549 as triple negative, basal-like cancer cell lines. Cells were classified as completely tubular (I), tubular with few fragments (II), fragmented with few tubules (III) or completely fragmented (IV) according to representative images in Figure 2B. We found an important difference in the number of cells classified as having mostly tubular mitochondria (I) between NT and breast cancer cell lines. The MCF10A cell line showed the highest tubular mitochondria when compared to cancer cells, indicating an important role for mitochondrial fission in breast cancer. Importantly, we found cells with fragmented mitochondria (IV) as being the most heterogeneous population among the cell lines, with a low percentage of fragmented mitochondria in MCF10A and a great diversity among the cancer cell lines studied. An image of the most representative mitochondrial morphology found in each cell line is shown in Figure 2C.

We selected genes in mitochondrial gene signatures related to mitophagy, mitochondrial dynamics and biogenesis to evaluate differences in mitochondrial function among breast cancer subtypes. We found differences in the expression level of genes related to mitochondrial biology in tumors from the TCGA sample set according to the molecular classification of tumors (Table 1, Supplementary Figure 1). Significant changes in the expression level of genes related to mitochondrial dynamics were observed among breast cancer subtypes. Fusion-related (OPA1, MFN1) as well as fission related (DNM1L) genes were found to be increased in the basal-like subtype when compared to Luminal A tumor samples. However, adaptor proteins for Drp-1 on the outer mitochondrial membrane like MID49/MIEF2 or FIS1 (Table 1, Supplementary Figure 1), were found to be decreased in the basal-like subtype when compared to the other breast cancer subtypes, and no changes among the different subtypes were observed in MFF, another Drp-1 adaptor protein (Table 1). Importantly, decreased levels of mitophagy-related BNIP3L and PINK1, as well as increased levels of mitochondrial biogenesis related genes PPARGC1A and PPARGC1B were found in the basal-like tumor samples when compared to the Luminal A subtype (Table 1, Supplementary Figure 1). This evidence suggests changes in mitochondrial quality control mechanisms, turnover and important differences in mitochondrial biology among breast cancer subtypes.

**Table 1:** Breast cancer subtypes show differences in the expression level of mitochondria-related genes.

		**Normal-like**		**Luminal A**		**Luminal B**		**HER2-enriched**		**Basal-like**	
	**Gene**	**Mean** ± **SD**	**ANOVA**	**Mean ± SD**	**ANOVA**	**Mean ± SD**	**ANOVA**	**Mean ± SD**	**ANOVA**	**Mean ± SD**	**ANOVA**
Fusion	OPA1	−0.25 ± 0.9		−0.244 ± 0.96		0.34 ± 1.3	1	0.978 ± 1.3	0,1,2	1.303 ± 1.5	0,1,2
	MFN1	−0.74 ± 1.18		−0.015, 1.15		0.516 ± 1.2	1	0.617 ± 1.4	0,1	1.117 ± 1.5	0,1,2
	MFN2	−0.17 ± 0.82		−0.124 ± 1.08		−0.54 ± 1.3	1	−0.57 ± 1.4		−0.56 ± 1.4	
Fission	DNM1L	−0.62 ± 1.04		−0.302 ± 1.03		0.276 ± 1.1	1	0.293 ± 1.2	1	1.023 ± 1.5	0,1,2,3
	MTP18/MTFP1	−0.28 ± 0.86		−0.585 ± 1.05		−0.01 ± 1.1	1	0.201 ± 1	1	0.235 ± 1.1	1
	YME1L1	−0.79 ± 1.77		−0.204 ± 1.1		0.378 ± 1.1	1	0.2 ± 1.4		0.843 ± 1.9	0,1
	MID51/MIEF1	−0.38 ± 0.75		−0.681 ± 1.05		−0.56 ± 1.3		0.297 ± 1.4	1,2	0.629 ± 1.2	1,2
	MARCH5	−1.47 ± 1.25		−0.14 ± 0.87	0	−0.09 ± 1.1	0	0.142 ± 1.3	0	−0.43 ± 1.3	3
	MFF	−0.29 ± 1.26		−0.119 ± 1.01		−0.23 ± 1		−0.17 ± 1.2		0.071 ± 1.3	
	FIS1	−0.61 ± 1.13		0.25 ± 0.97		0.151 ± 1.1		−0.44 ± 1	1,2	−0.82 ± 1	1,2
	OMA1	−0.29 ± 0.87		0.241 ± 0.95		0.115 ± 1.1		−0.66 ± 1.3	1,2	−0.56 ± 1	1,2
	MID49/MIEF2	−0.49 ± 0.79		0.11 ± 0.95		−0.28 ± 1.1		−0.39 ± 0.8	1,2	−1.24 ± 1	1,2,3
Mitophagy	BNIP3L	−0.5 ± 1.09		−0.084 ± 1.06		−0.76 ± 1.1	1	−0.75 ± 1.2	1	−1.33 ± 1.1	1, 2, 3
	PINK1	0.153 ± 0.93		0.146 ± 0.92		−0.44 ± 1	1	−0.51 ± 1	1	−0.84 ± 1.1	1
	PARK2	−0.5 ± 1.09		−0.084 ± 1.06		−0.76 ± 1.1	1	−0.75 ± 1.2	1	−1.33 ± 1.1	
Biogenesis	PPARGC1A	0.388 ± 0.39		−0.066 ± 0.7		−0.51 ± 0.7	1	0.278 ± 1	2	0.961 ± 1.6	1,2,3
	PPARGC1B	0.395 ± 0.87		−0.285 ± 0.84		−0.16 ± 1		0.531 ± 1	1,2	1.001 ± 0.9	1,2,3
Other	SIRT3	−1.04 ± 0.7		0.382 ± 0.93	0	−0.06 ± 1.1	0, 1	−0.82 ± 0.9	1,2	−1.4 ± 0.9	1,2,3

*mRNA expression z-scores (microarray) analyses from TGCA samples according to their breast cancer sybtype (PAM50 classification). Gray cell means differences with p < 0.05 to Normal-like (0), Luminal A (1), Luminal B (2), or HER2 (3)*.

### TNBC Was Characterized by an Increased Oxidation State

**Figure 3:**
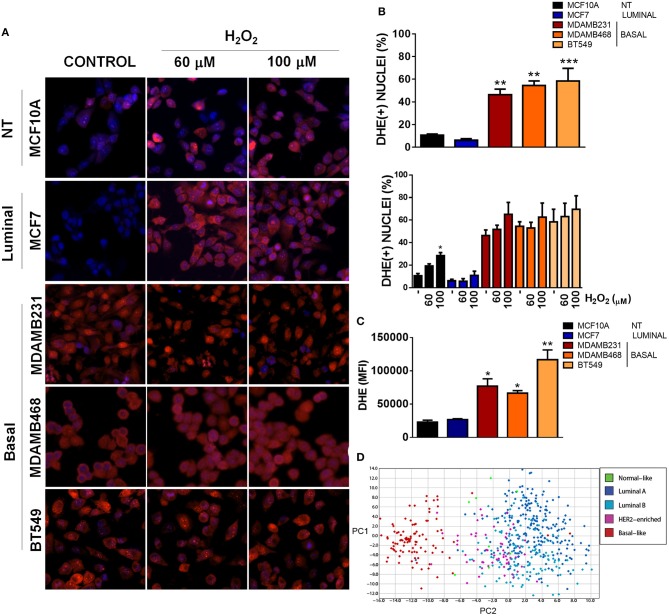
Basal, TNBC cell lines have elevated ROS levels when compared to non-tumorigenic or luminal breast cancer cell lines. **(A)** ROS levels were evaluated by fluorescence microscopy using dihydroethidium (DHE) staining (red) and total nuclei were stained with Hoechst (blue). Cells were treated with the indicated concentrations of H_2_O_2._ **(B)** DHE positive nuclei were counted and graphed as a percentage of total nuclei. **(C)** ROS levels were quantitatively evaluated by flow cytometry in the different cell lines. **(D)** A ROS-related gene signature PCA analysis clearly clustered basal-like tumors separate from the other breast cancer subtypes. Graphs show mean ± SEM of 3–5 independent experiments, *Different to MCF10A and MCF7 or to their respective control with *p < 0.05; **p < 0.01, and ***p < 0.001. PC, principal component.

Mitochondria have a crucial role in triggering redox signaling through ROS release from the electron transport chain (ETC) and ROS production is one of the aspects that has been involved in the promotion of malignancy by mitochondrial dysfunction (17). In this regard, ROS generation from mitochondria or from other cellular sources can contribute to the initiation of cancer in normal or non-malignant cells. Moreover, once a cell is transformed, redox signaling can amplify the malignant phenotype in terms of proliferation, survival, and migration through the activation of pro-tumorigenic signaling pathways (17, 28). Since we were particularly interested in TNBC, we analyzed ROS levels in the different TNBC cell lines under basal conditions using dihydroethidium (DHE) staining and compared to an ER+ and a non-tumorigenic control (Figures 3A–C). DHE is widely used as a small-molecule fluorescent ROS probe which is oxidized to 2-hydroxyethidium in the presence of ${\text{O}}_{2}^{\cdot -}$ and to ethidium enzymatically or in the presence of 1-electron oxidants, which reflect total oxidant generation (29, 30). Since both oxidation products are fluorescent in red and are not distinguishable in intact cells with the methods that we used (fluorescence microscopy and flow cytometry), we used DHE staining as a measurement of total ROS levels.

We found increased DHE(+) nuclei in basal-like, TNBC cell lines with more than 45% DHE positive nuclei in the three TNBC cell lines studied (MDAMB231, MDAMB468 and BT549) when compared to the luminal (MCF7) or non-tumorigenic (MCF10A) cell lines (Figures 3A,B). Interestingly, H_2_O_2_ treatment increased DHE positive nuclei only in the non-tumorigenic, MCF10A cell line, probably indicating a better redox balance in cancer cells or a high oxidation status in TNBC cells that cannot be further increased by oxidants. Also, flow cytometry analysis of DHE mean fluorescence intensity in each cell line showed increased DHE staining in basal-like, TNBC cell lines when compared to the non-tumorigenic MCF10A or the ER+/luminal MCF7 cells (Figure 3C). Finally, expression analysis of a ROS gene signature in tumor samples from the TCGA dataset clearly separated basal-like tumors from the other breast cancer subtypes, both when analyzed by unsupervised hierarchical clustering (Supplementary Figure 2) or principal component analysis (PCA, Figure 3D), indicating that oxidant and anti-oxidant gene expression is similar among basal-like tumor samples and different to other breast cancer subtypes. Thus, TNBC cells had a high level of oxidation when compared to cell lines from other subtypes, which could not be further increased with H_2_O_2_ treatment.

### Mitochondrial ROS Sustain Oncogenic Signaling and Survival in TNBC

**Figure 4:**
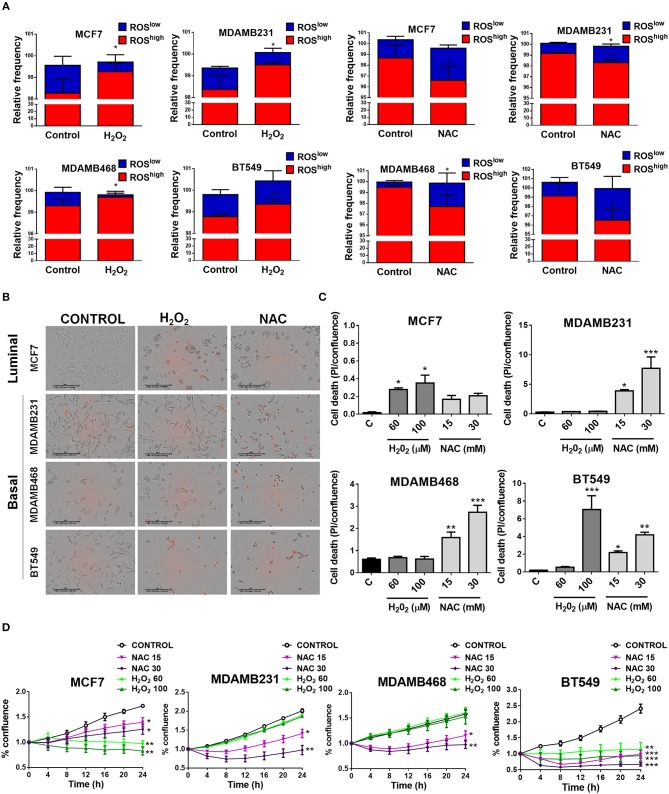
Basal, TNBC cell lines are dependent on ROS for survival. **(A)** Cells were treated with oxidating conditions (H_2_O_2_) or with an antioxidant (NAC, N-acetyl cysteine) at the indicated concentrations. One hundred micromolar H_2_O_2_ treatment increased the ROS^high^ or decreased the ROS^low^ population and 30 mM NAC had the opposite effect in all breast cancer cell lines studied. **(B,C)** Cell death was evaluated as propidium iodide staining [% confluency of PI(+) cells] and normalized to total % confluency. **(D)** Cell proliferation was evaluated as changes in cell confluency in an Incucyte real time cell imaging system. Graphs show mean ± SEM of more than 3 independent experiments. *different to control, *p* < 0.05; **different to control, *p* < 0.01; and ***different to control, *p* < 0.001.

To test if elevated ROS levels sustained oncogenic signaling and survival of TNBC cells, we used H_2_O_2_ to induce oxidation or the antioxidant N-acetyl cysteine (NAC) to decrease basal ROS levels in TNBC cell lines and compared to an ER+ cell line (MCF7) with low oxidation levels (Figure 4A). H_2_O_2_ treatment increased the ROS^high^ population and/or decreased the relative frequency in the ROS^low^ population in the cancer cell lines studied. NAC treatment decreased the relative frequency of the ROS^high^ or increased the ROS^low^ population (Figure 4A). We measured cell confluency as a measure of cellular viability and cell death using the cell impermeable dye propidium iodide (PI). Dead cells with compromised plasma membrane are permeable to the dye and red nuclei represent dead cells (Figures 4B,C). The MCF7 luminal breast cancer cell line was sensitive to H_2_O_2_ treatment since increased PI staining as well as decreased proliferation was observed (Figures 4B–D), whereas with NAC treatment, only proliferation was affected (decreased, Figure 4D) and no induction of cell death was observed (Figures 4B,C). Interestingly, TNBC cell lines (MDAMB231, MDAMB468, and BT549) showed the most striking changes in cell morphology, increased cell death, and decreased proliferation after antioxidant treatment (Figures 4B–D). Surprisingly, H_2_O_2_ had no effect on cell death (Figures 4B,C) or cell proliferation (Figure 4D) on MDAMB231 and MDAMB468 TNBC cell lines and both NAC and H_2_O_2_ induced cell death in the BT549 TNBC cell line. These results suggest that high ROS levels are responsible for maintaining oncogenic signaling in TNBC cells.

**Figure 5:**
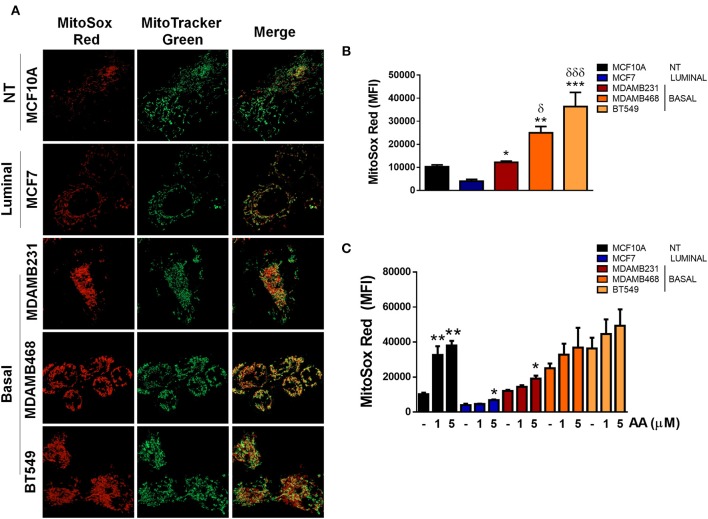
ROS in basal, TNBC cell lines are derived from the mitochondria. **(A)** Mitochondria were stained with Mitotracker (green) and MitoSox (red) to evaluate production of mitochondrial ROS. **(B)** Quantification of MitoSox fluorescence was performed by flow cytometry in basal conditions or **(C)** in cells treated with Antimycin A (AA) at the indicated concentrations. Graphs show mean ± SEM of 3 independent experiments. In **(B)** *different to MCF7 and ^δ^different to MCF10A. In **(C)** *different to control. *p < 0.05; **p < 0.01; ***p < 0.001; *δ <0.05; and ***δδδ <0.001.

In order to evaluate the source of ROS production, we explored if elevated ROS in TNBC cell lines were derived from mitochondria by co-staining cell lines with MitoSox (red) to evaluate mitochondrial ROS production and MitoTracker (green) to stain mitochondria and compared to ER+ MCF7 or non-tumorigenic MCF10A cell lines (Figure 5A). MitoSox is a DHE derivative with an additional cationic triphenylphosphonium group (TPP+). Due to its positive charge, MitoSox preferentially accumulates within the mitochondrial matrix and its red fluorescent oxidation products have been used to measure mitochondrial ROS production (31).

We found increased MitoSox staining in all the TNBC cell lines studied as well as increased co-localization of both stains indicating higher mitochondrial ROS production in the TNBC cell lines (Figure 5A). In addition, MitoSox levels were higher in the TNBC cell lines when compared to the luminal MCF7 cell line when MitoSox fluorescence intensity was quantified by flow cytometry (Figure 5B). Importantly, antimycin A (AA), a mitochondrial complex III inhibitor, only increased MitoSox fluorescence in the MCF10A, MCF7, and MDAMB231 cell lines, probably indicating that MDAMB468 and BT549 cell line mitochondria were producing high mitochondrial ROS in basal conditions that cannot be further increased by AA treatment (Figure 5C). Although the MDAMB231 TNBC cell line had increased MitoSox levels when compared to the MCF7 cell line, it showed high DHE fluorescence (Figure 3C) and its DHE fluorescence intensity was similar to MDAMB468 cells. Furthermore, frequency of MitoSox^high^ cells was lower than the DHE^high^ population in both the MDAMB231 and MDAMB468 but not in the BT549 cell line (Supplementary Figure 4). The previous data indicate that the MDAMB231 and the MDAMB468 cell line, to a lesser extent, have active ROS sources additional to the mitochondria that contribute to the maintenance of their high oxidation state.

## Discussion

Alterations in mitochondrial dynamics and function have been related to malignancy in different types of cancer. In breast cancer, increased mitochondrial fission-related Drp1 protein levels have been found in breast carcinomas and lymph node metastases (25), increased mitochondrial fission has been observed in TNBC cell lines and it has been shown that mitochondrial fission is necessary for cell migration and invasion (25). It has also been shown that invasive breast cancer cells have increased oxidative phosphorylation (OXPHOS), mitochondrial biogenesis, and oxygen consumption rates when compared to their non-invasive counterparts (32). Differences in mitochondrial mass have been observed in primary human breast tumors (33), and mitochondria from breast cancer cell lines with different metastatic capacities have been shown to have different functional characteristics (34). In agreement with the previously published reports, a mitochondria-related gene signature clustered breast cancer tumor samples according to their intrinsic subtype (Figure 1) and we found important differences in mitochondrial morphology when comparing the non-tumorigenic MCF10A with all the breast cancer cell lines tested (Figures 2A,C). Breast cancer cell lines had more fragmented mitochondria than the non-tumorigenic cell line, which had almost 40% of cells with a tubular morphology (I) while all the cancer cell lines had < 15% indicating an important role for mitochondrial fragmentation in transformation. However, despite significant differences in the expression levels of genes related to mitochondrial dynamics among breast tumor subtypes (Table 1, Supplementary Figure 1), we did not find a clear relationship between mitochondrial morphology and breast cancer subtype in the cell lines studied. Although luminal cell lines MCF7 and T47D showed a very similar mitochondrial morphology, mitochondrial shape from basal, TNBC cell lines was highly heterogeneous (Figure 2A). Nevertheless, the basal-like subtype was the most different to other breast cancer subtypes with regards to changes in mitochondria-related gene expression (Table 1, Figure 1), suggesting mitochondrial alterations different to mitochondrial shape. So, although protein levels of individual mitochondrial-dynamics related proteins (e.g., Drp1) have been related to increased levels of malignancy (25), the global changes in mitochondria-related gene expression that we studied (Figure 1), clustered breast cancer samples according to tumor subtypes and seem to be related to changes in mitochondrial function rather to a specific mitochondrial morphology. Indeed, we found increased DNM1L/Drp1 mRNA levels in Basal-like breast cancer samples (Table 1, Supplementary Figure 1) but we also found increased mitochondrial fusion and biogenesis-related gene expression, as well as decreased mitophagy-related genes, suggesting global changes in mitochondrial function beyond mitochondrial morphology. Importantly, changes in mitochondrial gene expression, particularly of genes related to mitochondrial dynamics could also reflect a distinct association of mitochondria with other organelles, like the endoplasmic reticulum, which has been shown to regulate cellular processes like endoplasmic reticulum stress, autophagy and inflammasome signaling (35), but this was not evaluated in this study.

One of the consequences of mitochondrial disfunction that has been involved in several aspects of carcinogenesis is the production of ROS and elevated ROS levels have been found in tumor cells from different tissues (14, 36). In this regard, it has been proposed that cancer cells are able to modulate their antioxidant capacity to achieve a different redox balance than normal cells. In this setting, overproduction of ROS due to oncogenic signaling or metabolic alterations has been shown to result in increased antioxidant capacity that is able to maintain oncogenic ROS signaling, allowing disease progression and avoiding cell death (28). Oncogenic signaling pathways that are known to be activated by ROS include the NF-kB (37), NRF2, Wnt (38), and EGFR signaling pathways (39, 40). Also, ROS have been shown to activate the tumor suppressor p53 and mediate apoptosis (41). Importantly, basal-like tumors are known to present TP53 loss of function in most, if not all, tumors and show amplification of the RAS-RAF-MEK pathway including amplifications in EGFR (7). We found increased ROS levels in TNBC cell lines when compared to a non-tumorigenic or an ER+/luminal breast cancer cell line. The increased oxidation state was necessary for cell survival since antioxidant treatment induced cell death in TNBC cell lines and not in non-tumorigenic or an ER+/luminal breast cancer cell line. Our results demonstrate that increased ROS production in TNBC cell lines have a pro-tumorigenic role by sustaining the oncogenic signaling necessary for their proliferation and survival and suggest that the loss of function of p53 characteristic of this type of breast cancer might be necessary to survive this strongly oxidizing conditions. Mitochondrial ROS were the main source of ROS in TNBC cell lines (Figure 5) and mitochondrial ROS levels were related to mitochondrial shape (Figure 2), since MitoSox fluorescence correlated with the percentage of cells in the fragmented classification (IV, Figure 2A) for the TNBC subtype. Our results relate differences in mitochondrial shape among TNBC cell lines with mitochondrial ROS production and suggest that those TNBC cell lines with low levels of fragmented mitochondria (e.g., MDAMB231 cell line) could have additional ROS sources to maintain oxidative conditions and signaling, while those TNBC cells with high levels of fragmented mitochondria (BT549) would rely only on mitochondrial ROS production to sustain oncogenic signaling.

In the literature, there is conflicting evidence regarding the use of antioxidants during cancer progression and treatment. In normal cells or pre-cancerous lesions, ROS have been proposed to induce DNA damage and increase oncogenic mutations, raising the possibility that dietary supplementation with antioxidants could suppress the initiation or progression of some types of cancer. However, antioxidant treatment is known to suppress cancer initiation in some contexts and increase cancer progression in others (16). Moreover, the use of dietary antioxidants has not been shown to reduce cancer incidence and in fact, antioxidant supplementation has actually increased incidence and death from some types of cancer including lung cancer (42) or increase the risk of developing another type of unrelated diseases (43, 44). In cancer progression models there is also contradictory evidence regarding the use of antioxidants for the treatment of cancer. For instance, in a melanoma mouse model, metastatic cells had higher cytoplasmic and mitochondrial ROS levels and lower mitochondrial mass. The authors proposed that the high levels of oxidation in metastasis are limiting for the establishment of metastasis, since the treatment with antioxidants increased the number of circulating tumor cells and metastatic burden without affecting the growth of the tumor (45). On the other hand, in another study, highly metastatic cells were found to have increased mitochondrial activity and superoxide production. In this case, antioxidant treatment decreased migration and invasiveness which was proposed to be due to ROS-mediated Src activation in tumor cells (46). In breast cancer, in the PyMT mouse cancer model, mice with decreased glutathione content due to deficiency in GCLM (glutamate cysteine ligase modifier), a subunit of glutamate cysteine ligase, necessary for glutathione synthesis or mice treated with BSO (buthionine-[S,R]-sulfoximine) to chemically inhibit glutathione synthesis, had decreased mammary tumor burden. When formed, tumors in PyMT-Gclm-/- mice, showed reduced proliferation and progression (47). So, at least in this model, pre-cancerous lesions need the antioxidant effect of glutathione to progress to breast cancer, which would argue against the use of antioxidants for breast cancer treatment. On the other hand, also in breast cancer, antioxidant treatment decreased DNA lesions and tumorigenesis in a murine model of BRCA1-deficient, p53^+/−^ breast cancer, where excessive estrogen metabolism increased cancer cell ROS production and DNA damage (48). Also, ROS scavenging by overexpression of exogenous EcSOD (extracellular superoxide dismutase), decreased invasion of breast cancer cells *in vitro* (49), and decreased metastasis *in vivo* in TNBC mouse models (50). Moreover, increased ROS levels have been associated with BRCA1 mutations in this type of breast cancer (40).

In agreement with the previous studies, where BRCA1 mutations, which predispose to TNBC have been found to have increased ROS levels, our data shows increased ROS levels in all the TNBC cell lines studied in comparison to an ER+ breast cancer cell line or the non-tumorigenic cells. Moreover, high ROS levels seem to be necessary for the maintenance and survival of this type of cancer since antioxidant treatment greatly decreased proliferation and induced cell death. Our results suggest a potential use for ROS or oxidation products in cancer cells as biomarkers of malignancy in TNBC which is currently diagnosed by the absence of immunohistochemistry biomarkers. Furthermore, mitochondrial ROS could function as a therapeutic target against this cancer subtype, which currently lacks a targeted therapy. Our data explains controversies in the literature regarding the use of antioxidants for cancer therapy and we propose that antioxidant treatments should only be used in those cancer cells with high basal ROS levels which are likely to use this pro-oxidant conditions to sustain oncogenic signaling. Our results also suggest that a similar approach could be used in those types of cancer which have been shown to have increased oxidation levels or similar mechanisms of transformation as TNBC (e.g., *BRCA1* inactivation, *RB1* loss, *TP53* inactivation or amplifications in the *MAPK* pathway) (7). In this regard, serous ovarian carcinomas have been shown to have a similar mutation and expression profile as TNBC (7) and has also been shown to manifest a pro-oxidant state (51), indicating a potential use for antioxidant treatment in this type of cancer. Other cancer types that have also been characterized by increased oxidation levels and in which a role of ROS has been proposed in the promotion of malignancy include prostate (52), gastric (53, 54), and pancreatic cancer (55). Importantly, our data also suggests that antioxidant treatment should not be suggested as a general therapy for cancer in these tissues since heterogeneity in tumors from those tissues has also been reported (56–58) and a careful analyses of those cancer subtypes which utilize pro-oxidant signaling for survival should be performed.

Our data also suggests a possible explanation for the anti-cancer effect of drugs with yet unclear mechanisms of action like metformin. This drug has been shown to have anti-cancer effects on breast (59) and other types of cancer (60, 61), has been proposed to have an antioxidant effect (62), and is known to be particularly effective on the TNBC subtype (63). So, our results indicate a potential use for this drug or for antioxidant nutraceuticals (64) for the treatment of this type of cancer and for cancers with similar mechanisms of oncogenicity involving increased oxidation states.

Finally, our results underscore the role of mitochondrial ROS production in sustaining oncogenic signaling in the basal, TNBC subtype as the main, but likely not only, source of ROS and highlights their potential use as a therapeutic target in this breast cancer subtype with current limited therapeutic options.

## Data Availability

All datasets generated for this study are included in the manuscript and/or the Supplementary Files.

## Author Contributions

FS-S, PC-H, RP, LM-P-P, and PM contributed conception and design of the study. FS-S, AD-M, JM-A, DR-R, and JF-A performed the experiments. FS-S, JR-L, IH-C, MA-R, RP, and PM wrote sections of the manuscript. All authors contributed to manuscript revision, read and approved the submitted version.

### Conflict of Interest Statement

The authors declare that the research was conducted in the absence of any commercial or financial relationships that could be construed as a potential conflict of interest.
