# Supplementary figures and images for: Breast Cancer Subtypes Present a Differential Production of Reactive Oxygen Species (ROS) and Susceptibility to Antioxidant Treatment
Source: Front Oncol. 2019 Jun 7;9:480. doi: 10.3389/fonc.2019.00480 (PMC6568240; doi:10.3389/fonc.2019.00480)

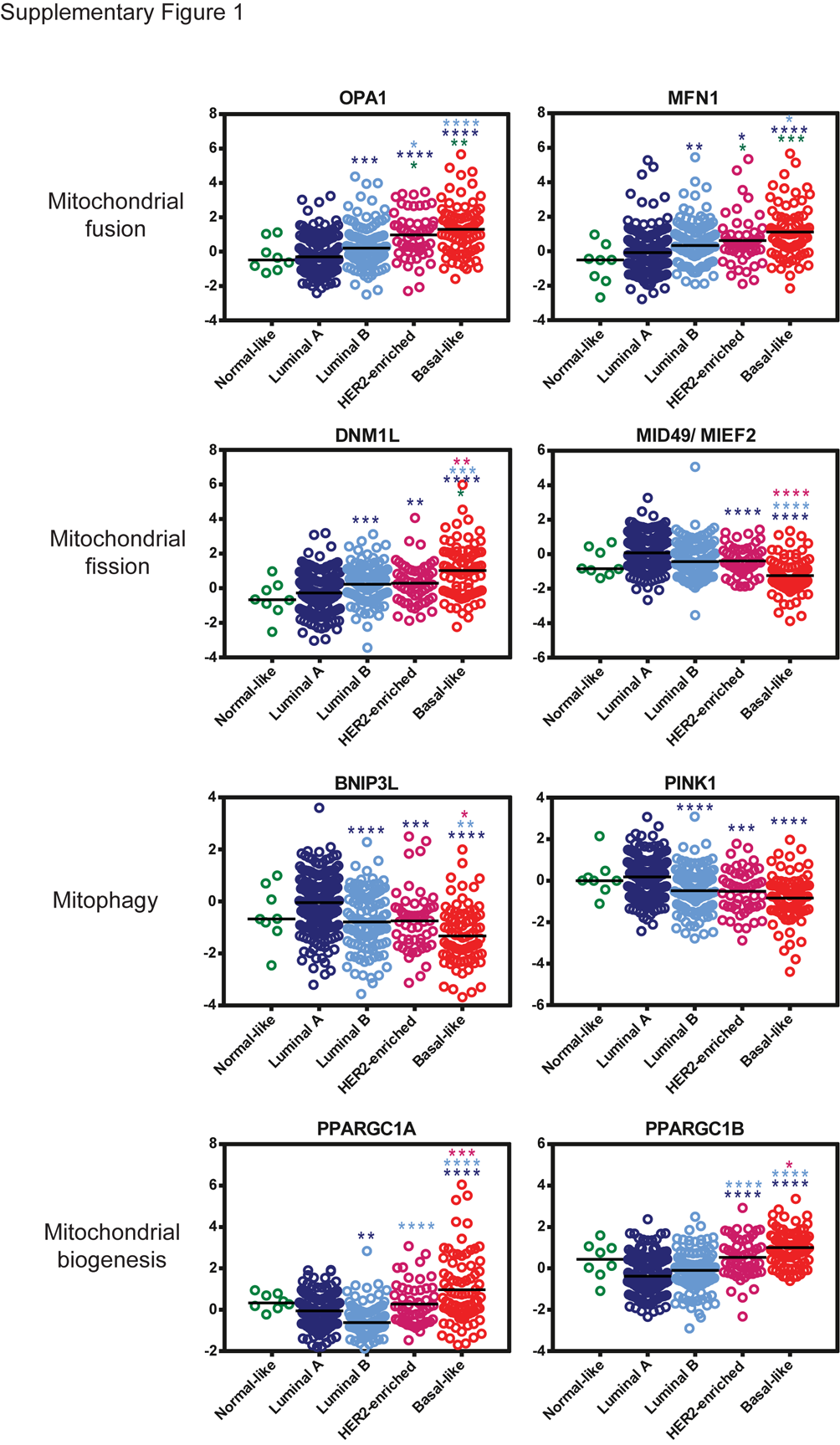

Supplement: Supplementary file 1 [file Image_1.TIF]

Supplementary Figure 2

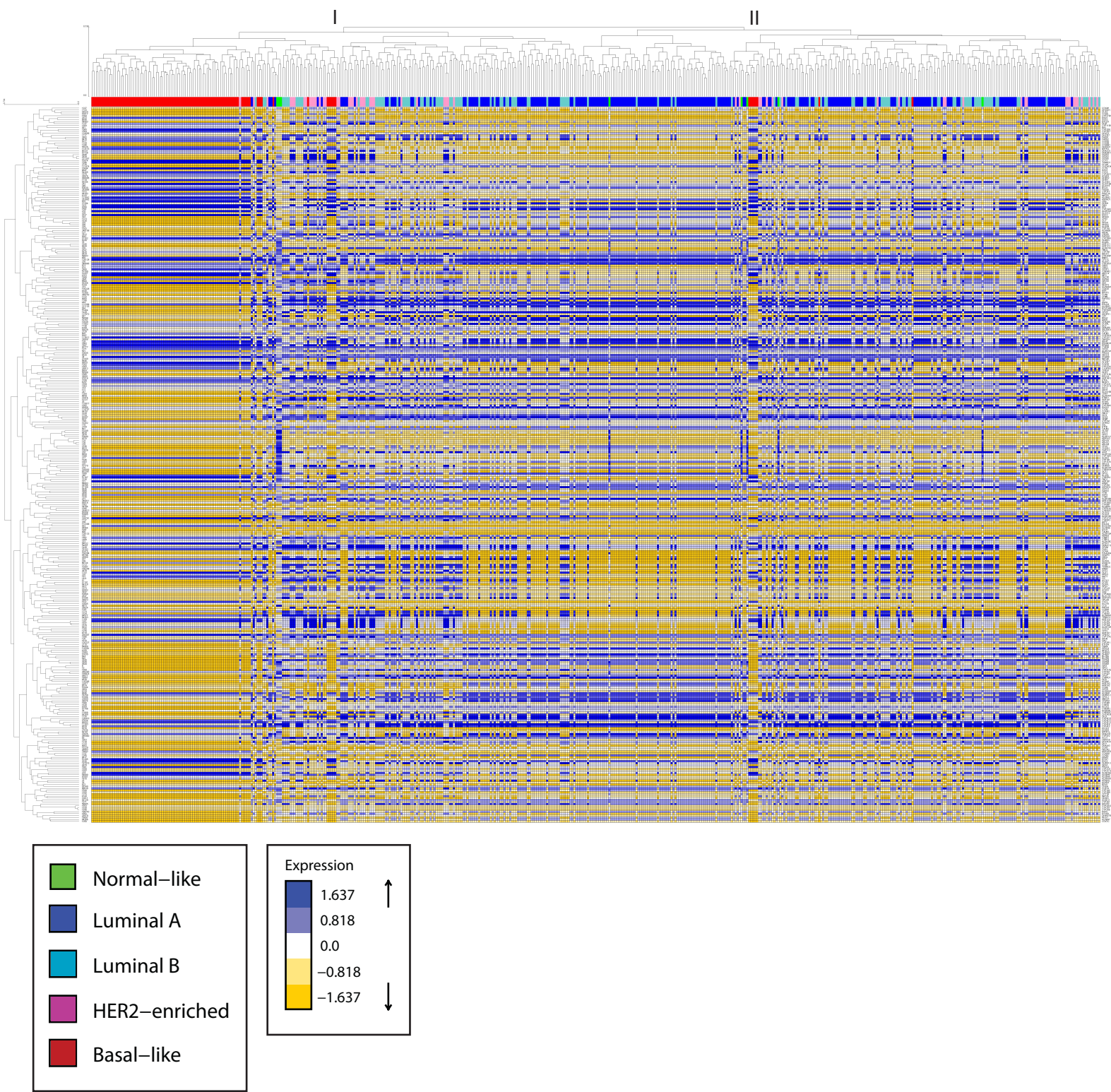

Supplement: Supplementary file 2 [file Image_2.pdf]

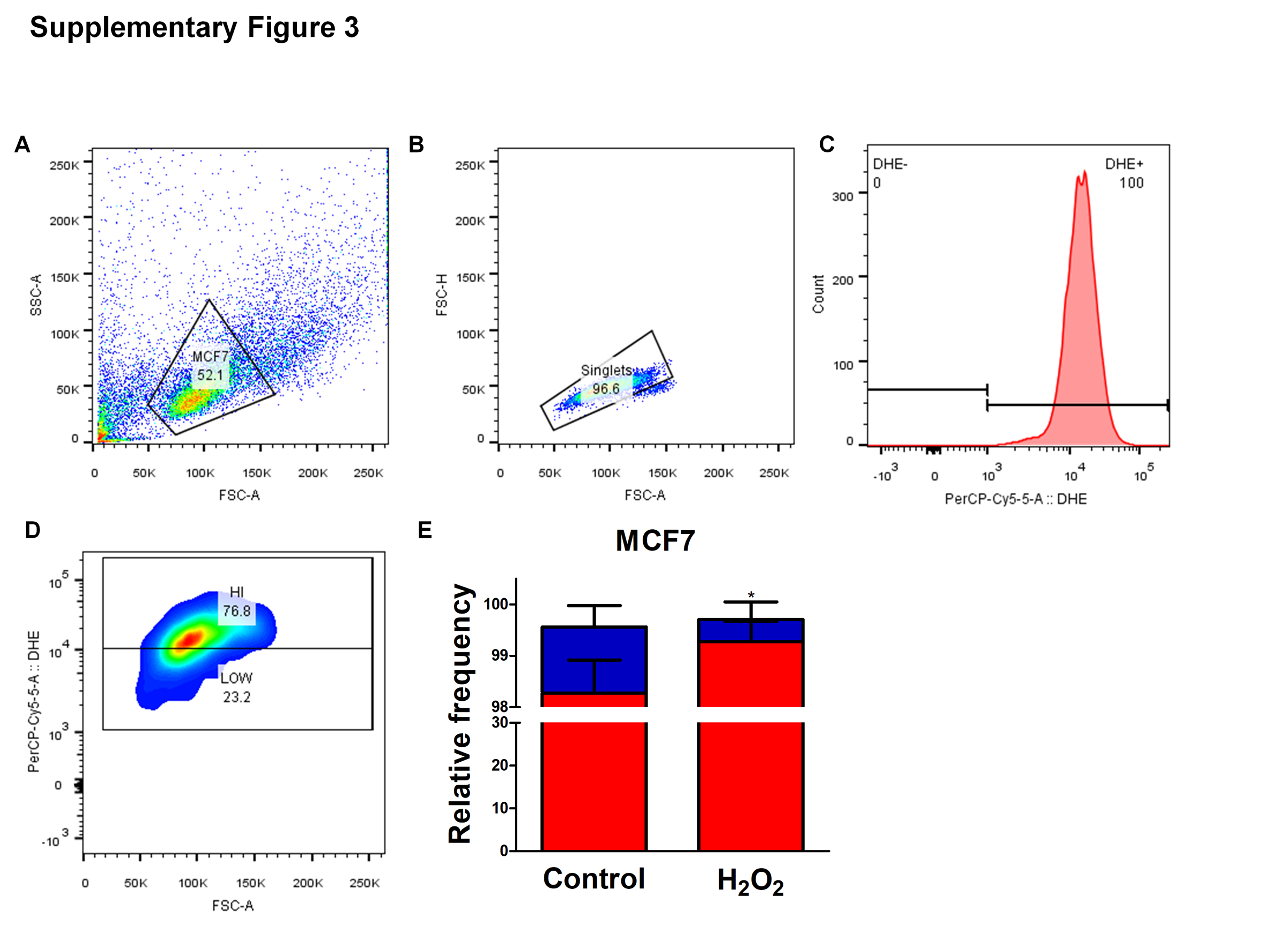

Supplement: Supplementary file 3 [file Image_3.tif]

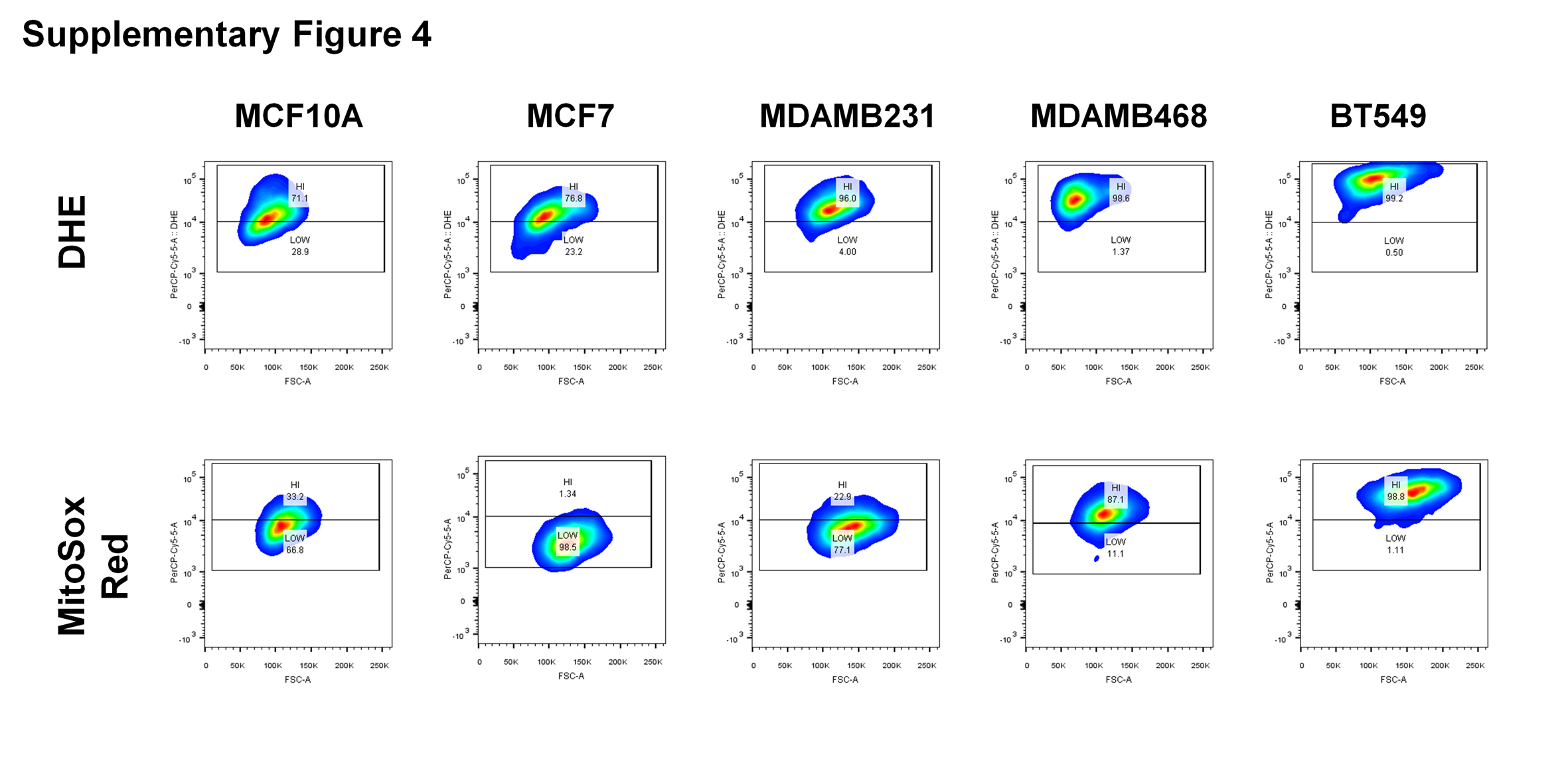

Supplement: Supplementary file 4 [file Image_4.tif]
