# Supplementary material for: Breast Cancer Subtypes Present a Differential Production of Reactive Oxygen Species (ROS) and Susceptibility to Antioxidant Treatment
Source: Front Oncol. 2019 Jun 7;9:480. doi: 10.3389/fonc.2019.00480 (PMC6568240; doi:10.3389/fonc.2019.00480)
Supplement: Supplementary file 5 [file Table_1.DOCX]

Supplementary Material

# Supplementary Figures and Tables

## Supplementary Figures

**Supplementary Figure 1. Breast cancer subtypes show differences in the expression levels of mitochondria-related genes.** Selected genes related to mitochondrial dynamics, mitophagy or mitochondrial biogenesis were chosen from Table 1 and their mRNA expression Z-score was graphed according to the breast cancer subtype (PAM50 classification). Z-score was obtained from cbioportal.org from TGCA samples. The graphs show mean expression per breast tumor subtype analyzed. Z-scores were analyzed using an ANOVA. **** p<0.0001; *** p<0.001; ** p<0.01; * p<0.05; color indicates difference to the respective subtype.

**Supplementary Figure 2. A ROS gene signature separates Basal-like TNBC tumor samples from tumors of other breast cancer subtypes in a bioinformatic analysis of gene expression.** ROS-related gene expression was analyzed in TCGA breast tumor samples using unsupervised hierarchical clustering analysis, revealing clusters of samples from the different molecular subtypes. Two major clusters were found (I and II). Cluster I was enriched in basal-like tumors together with some HER2-enriched samples and a sub-cluster containing luminal samples, mostly luminal B samples. Cluster II was enriched in luminal A tumors, with some luminal B, HER2- enriched and a few basal-like tumors.

**Supplementary Figure 3. Flow cytometry analysis pipeline for ROS^high^ and ^low^ populations.** (A) For ROS^high^ and ^low^ population analysis, after singlet and event selection, (B) DHE+ cells were analyzed. (C and D) Gates for ROS^high^ and ^low^ populations were established according to H_2_O_2_ treatment, where the relative frequency of ROS^high^ population significantly increased after H_2_O_2_ treatment in each cell line.

**Supplementary Figure 4. DHE and MitoSox contour plots for all the breast cancer cell lines.** For comparison of total ROS with mitocondrial ROS production, DHE and MitoSox staining are shown in the same figure.

**Supplementary Table 1. Mitochondria-related genes and ROS-related genes.**
